# Looked after children and access to dental services and oral health in Scotland: a national data-linkage study (LAC-DENTAL)

**DOI:** 10.23889/ijpds.v1i1.324

**Published:** 2017-04-19

**Authors:** Alex McMahon, Katharine Sharpe, Lorna Macpherson, Rachael Wood, Graham Connelly, Milligan Ian, Philip Wilson, David Clark, Albert King, Lawrie Elliott, David Conway

**Affiliations:** 1 University of Glasgow Dental School; 2 NHS National Services Scotland; 3 University of Strathclyde; 4 University of Aberdeen; 5 Education Analytical Services, Scottish Government; 6 Edinburgh Napier University

## Objectives

Children that are `looked after' include those that are accommodated in foster, kinship and residential care placements, as well as those at home on compulsory supervision. They have poorer physical and mental health than their peers and there are concerns about the relatively high levels of untreated morbidity. Oral health and access to dental services among Looked After Children (LAC) has received limited attention to date. The objective of this study was to compare the oral health and access to dental services of children who are looked after by the state, with comparable children in the general population. 

## Approach

School and Social Work datasets were able to be linked using the Scottish Exchange of Data (ScotXed) Unit. This in turn was linked with health data making use of the Scottish national record-linkage system provided by the NHS eDRIS team for the FARR Institute Scotland. All of the following datasets used in this study are complete national datasets for the time periods noted.

School Pupil Census 2012: a census of children in local authority primary and secondary schools that provides each child’s age, sex and socioeconomic status as measured by the Scottish Index of Multiple Deprivation (SIMD). The other datasets cover the period 2008-2012: LAC- all children with social work referrals for various types of placement; registration with dentists; hospital discharge data for all episodes of tooth extraction; National Dental Inspection Programme (NDIP) data from Primary 1 and Primary 7 school years (dental decay). The LAC group were compared to their peers by logistic regression adjusted by SIMD, using remote access to the National Safe Haven. 

## Results

There were 633204 subjects in the study group (10927 LAC, 622280 nonLAC). Ages ranged from four to 17 years (mean 12 LAC and 10 nonLAC); with 53% male for LAC and 51% nonLAC; and 42% in the most deprived SIMD level for LAC and 21% for non-LAC. The subjects in the LAC group were more likely to have dental decay at Primary 1, odds-ratio (OR) 2.93 (2.55, 3.38), and Primary 7, OR 1.81 (1.68, 1.94). LAC subjects were less likely to be registered with a dentist, OR 0.50 (0.43, 0.59), and more likely to have teeth extracted, OR 1.50 (1.40, 1.60). All tests p<0.001.

## Conclusions

Looked after children are more likely to have dental problems and less likely to use dental services than their peers, after adjustment for socioeconomic status.

